# Supplementary material for: Caterpillars on a phytochemical landscape: The case of alfalfa and the Melissa blue butterfly
Source: Ecol Evol. 2020 Apr 21;10(10):4362–74. doi: 10.1002/ece3.6203 (PMC7246198; doi:10.1002/ece3.6203)
Supplement: Supplementary file 1 — Figures S1‐S8 and Tables S1‐S3 [file ECE3-10-4362-s001.pdf]

Supporting Information for:

"Caterpillars on a phytochemical landscape: The case of alfalfa and the Melissa blue butterfly" by Forister et al.

Contents:

Supporting Information Table S1: Details on individual compounds

Supporting Information Table S2: Results from lasso regression allowing for interactions

Supporting Information Figure S1: Correlations among modules, SLA, protein and leaf toughness

Supporting Information Figure S2a, S2b and S2c: Correlations within phytochemical modules

Supporting Information Figure S3: Variation among plants in phytochemical modules

Supporting Information Figure S4: Correlational structure among compounds, color coded by compound class identities.

Supporting Information Figure S5: Results from cross-validation and randomization experiments

Supporting Information Figure S6: Frequency and magnitude of interactions by compound class

Supporting Information Figure S7: Effects of specific compounds compared to coefficients of variation

Supporting Information Figure S8: Illustrations to accompany description of relative mass defect method.

TABLE S1 Details on individual compounds (numbered along the left column), including module numbers (see Figure 2), chemical class assignments, ridge regression beta coefficients (for caterpillar survival, development time and adult weight as in Figure 3), and mass spectra results: *m/z*, retention time (RT ave.) and relative mass defect (RMD). Compounds are organized by module number followed by compound number (an arbitrary designation).

Cpd.	Module	Class	Surv.	Dev.	Weight	*m/z*	RT ave.	RMD
65	m. 0	Phospholipid (PP)	0.00026	-0.1273	0.2072	299.2719	6.451733	909
180	m. 0	Sterol (St)	0.00067	-0.0684	0.0740	485.3591	27.309711	740
23	m. 1	Peptide (Pe)	0.00065	-0.0629	0.1109	492.2455	4.985533	499
30	m. 1	Alkaloid (Al)	0.00059	0.0172	-0.0105	366.1097	5.301200	300
35	m. 1	Saponin (Sa)	0.00020	0.0112	0.1319	459.2233	5.533244	486
37	m. 1	Peptide (Pe)	-0.00041	0.0073	0.1304	1564.7065	5.623800	452
50	m. 1	Peptide (Pe)	-0.00048	-0.0165	0.1683	1238.5847	5.966289	472
53	m. 1	Peptide (Pe)	-0.00042	-0.0301	0.0121	1106.5421	6.010844	490
57	m. 1	Peptide (Pe)	-0.00183	0.0547	0.1034	256.2642	6.141222	1031
58	m. 1	Peptide (Pe)	-0.00114	-0.0546	0.1899	1192.5417	6.211267	454
63	m. 1	Phospholipid (PP)	-0.00320	0.0857	-0.1410	518.3253	6.374000	628
64	m. 1	Peptide (Pe)	-0.00053	0.0427	0.0425	562.322	6.399089	573
67	m. 1	Phospholipid (PP)	-0.00246	-0.0880	0.0248	284.2959	6.617089	1041
70	m. 1	Peptide (Pe)	0.00052	-0.0476	0.1226	1168.6682	7.022178	572
78	m. 1	Peptide (Pe)	0.00060	0.0464	-0.0439	694.402	7.380578	579
84	m. 1	Phospholipid (PP)	0.00100	-0.0265	0.0137	293.2112	8.701933	720
89	m. 1	Phospholipid (PP)	-0.00032	-0.0165	0.0532	546.3438	10.332689	629
93	m. 1	Alkaloid (Al)	-0.00029	-0.0176	0.0149	358.2468	10.897978	689
95	m. 1	Lipid (Li)	0.00168	0.0453	0.0941	425.2903	11.479511	683
111	m. 1	Alkaloid (Al)	-0.00022	0.0119	0.0602	592.5035	15.707178	850
113	m. 1	Sterol (St)	0.00100	0.0130	-0.0143	661.3032	15.991800	458
128	m. 1	Peptide (Pe)	-0.00269	0.0354	-0.0869	584.4235	17.597156	725
129	m. 1	Peptide (Pe)	-0.00106	-0.0033	-0.0254	567.4209	17.818844	742
136	m. 1	Peptide (Pe)	0.00051	0.0017	0.0215	932.6342	18.698356	680
139	m. 1	Phospholipid (PP)	0.00205	0.0077	-0.0888	792.5648	18.853422	713
146	m. 1	Sterol (St)	-0.00004	-0.0499	-0.0437	451.3596	20.131378	797
149	m. 1	Phospholipid (PP)	-0.00047	0.0087	-0.0978	784.5599	20.769978	714
157	m. 1	Sterol (St)	0.00117	0.0258	0.0326	429.3741	21.812222	871
160	m. 1	Phospholipid (PP)	-0.00322	0.0023	-0.1929	747.6095	21.920444	815
161	m. 1	Phospholipid (PP)	0.00061	0.0140	-0.2701	552.4296	22.245667	778
162	m. 1	Phospholipid (PP)	-0.00307	0.0165	-0.1205	907.5367	22.382756	591
163	m. 1	Peptide (Pe)	-0.00153	-0.0016	-0.1119	886.5581	22.381711	630
164	m. 1	Peptide (Pe)	0.00277	-0.0499	0.1871	830.676	22.432867	814
165	m. 1	Lipid (Li)	0.00398	-0.0385	0.0233	537.5365	22.754333	998
167	m. 1	Phospholipid (PP)	0.00199	0.0207	0.0001	708.5134	23.041267	725
169	m. 1	Lipid (Li)	0.00100	0.0316	0.0242	565.5681	23.807289	1004
170	m. 1	Phospholipid (PP)	0.00214	-0.0376	-0.0473	885.5552	24.037489	627
171	m. 1	Lipid (Li)	0.00310	0.0220	0.0281	536.4387	24.087178	818
172	m. 1	Phospholipid (PP)	0.00194	-0.0314	-0.0176	869.559	24.216778	643
173	m. 1	Phospholipid (PP)	-0.00145	-0.1120	0.0485	787.427	24.262511	542
175	m. 1	Pigment (Pi)	0.00138	-0.0712	-0.1041	871.5766	24.897822	662
177	m. 1	Lipid (Li)	-0.00052	0.0206	0.0080	208.1347	27.036422	647
18	m. 2	Phen. glyco. (Pg)	-0.00246	-0.0574	0.0201	323.0738	4.163533	228
33	m. 2	Phen. glyco. (Pg)	-0.00040	-0.0210	-0.0409	493.1329	5.430044	505
69	m. 2	Peptide (Pe)	0.00001	-0.0226	-0.0657	1018.537	6.846578	527
92	m. 2	Lipid (Li)	0.00220	-0.0455	0.1122	375.2549	10.640356	679
94	m. 2	Peptide (Pe)	0.00041	-0.1458	0.0496	618.4291	11.238667	694
99	m. 2	Alkaloid (Al)	-0.00001	-0.0174	-0.0306	618.4291	12.470689	694
100	m. 2	Peptide (Pe)	0.00235	-0.0290	-0.0485	600.4193	13.376289	698
101	m. 2	Alkaloid (Al)	0.00040	-0.0528	0.0074	618.4291	13.049600	694
103	m. 2	Peptide (Pe)	-0.00012	-0.0534	-0.0377	603.4311	13.358222	714
104	m. 2	Peptide (Pe)	0.00242	-0.0386	-0.0329	600.4193	13.376289	698
109	m. 2	Peptide (Pe)	0.00025	-0.0441	0.0031	600.4193	15.398533	698
112	m. 2	Peptide (Pe)	-0.00036	0.0008	-0.0056	600.4193	15.860356	698

114	m. 2	Peptide (Pe)	0.00337	0.0671	0.0466	584.4235	16.288622	725
122	m. 2	Peptide (Pe)	0.00642	0.0166	0.1315	584.4235	16.758622	725
133	m. 2	Phospholipid (PP)	-0.00094	0.0888	-0.1533	716.5243	18.284733	732
134	m. 2	Peptide (Pe)	0.00260	0.0043	0.0275	568.4294	18.417311	755
135	m. 2	Peptide (Pe)	-0.00007	-0.0027	0.0696	937.5896	18.675222	629
154	m. 2	Sterol (St)	0.00435	-0.1104	0.0743	693.425	21.485978	613
155	m. 2	Phospholipid (PP)	0.00395	-0.0670	0.0774	635.3816	21.488511	601
166	m. 2	Pigment (Pi)	-0.00396	0.0522	-0.0076	871.5766	22.912311	662
168	m. 2	Peptide (Pe)	0.00091	-0.0479	0.0062	1057.7337	23.685733	694
2	m. 3	Halogen (Ha)	-0.00085	0.0590	0.0426	199.0946	0.546622	475
5	m. 3	Alkaloid (Al)	-0.00443	0.0446	-0.0332	217.0692	0.628311	485
6	m. 3	Peptide (Pe)	-0.00292	-0.0089	0.0744	309.1795	0.638667	581
7	m. 3	Sugar (Su)	-0.00441	0.0855	-0.1219	365.1066	0.653400	292
9	m. 3	Alkaloid (Al)	-0.00708	0.0404	-0.0229	321.1156	0.696467	360
12	m. 3	Alkaloid (Al)	-0.00114	0.0704	-0.1614	186.0765	0.831267	411
13	m. 3	Alkaloid (Al)	-0.00088	0.0796	-0.1770	140.071	0.843044	1008
14	m. 3	Peptide (Pe)	-0.00221	0.0548	-0.0971	225.0855	1.036511	380
34	m. 3	Phospholipid (PP)	-0.00396	-0.0466	-0.0396	547.3472	5.507867	634
38	m. 3	Saponin (Sa)	-0.00193	0.0677	-0.0541	633.348	5.635956	549
41	m. 3	Saponin (Sa)	-0.00335	-0.1640	0.1125	471.3488	5.634111	740
42	m. 3	Saponin (Sa)	-0.00151	0.0292	-0.1282	631.3333	5.725444	816
45	m. 3	Saponin (Sa)	-0.00020	-0.1281	0.1960	1105.5828	5.781400	527
55	m. 3	Saponin (Sa)	-0.00096	-0.1231	-0.1934	617.3527	6.043333	678
62	m. 3	Saponin (Sa)	-0.00139	-0.0482	-0.1629	825.4299	6.314022	521
120	m. 3	Phospholipid (PP)	-0.00122	-0.0073	-0.0314	732.5644	16.529933	770
141	m. 3	Phospholipid (PP)	-0.00302	-0.0398	0.0321	963.6051	18.994089	628
143	m. 3	Phospholipid (PP)	-0.00352	0.0180	0.0291	951.6048	19.629644	636
174	m. 3	Phospholipid (PP)	-0.00473	0.0474	-0.0259	805.6846	23.370044	759
27	m. 4	Phen. glyco. (Pg)	0.00157	-0.0575	0.1663	441.2984	5.217978	676
80	m. 4	Phospholipid (PP)	0.00323	-0.0657	0.1113	518.3253	7.756911	628
83	m. 4	Phospholipid (PP)	0.00205	0.0001	0.0979	520.3405	8.404400	654
85	m. 4	Phospholipid (PP)	0.00189	-0.0632	-0.0102	454.2938	8.763000	647
86	m. 4	Phospholipid (PP)	0.00389	-0.0043	-0.0528	496.3412	8.889378	687
91	m. 4	Phospholipid (PP)	0.00532	0.0847	0.1361	524.3727	10.499178	711
140	m. 4	Phospholipid (PP)	0.00237	-0.0266	-0.0049	797.5204	18.879244	653
142	m. 4	Phospholipid (PP)	-0.00099	0.0177	0.0110	714.5088	19.293533	711
144	m. 4	Phospholipid (PP)	-0.00135	0.0014	0.0366	756.5562	19.707467	735
148	m. 4	Phospholipid (PP)	-0.00123	0.0070	0.0349	716.525	20.684533	732
151	m. 4	Phospholipid (PP)	-0.00066	0.0324	-0.0100	801.5511	20.976333	688
153	m. 4	Phospholipid (PP)	-0.00212	0.0224	0.0440	758.5721	21.488156	754
159	m. 4	Phospholipid (PP)	0.00041	0.1169	0.0507	803.5978	22.069778	744
4	m. 5	Alkaloid (Al)	0.00060	-0.0718	0.0563	104.1077	0.595533	1035
8	m. 5	Alkaloid (Al)	0.00026	0.0377	0.0717	144.1024	0.663689	711
10	m. 5	Lipid (Li)	0.00096	0.0048	-0.0902	301.2133	0.700111	708
11	m. 5	Alkaloid (Al)	0.00122	-0.0359	-0.0768	158.1181	0.705733	747
15	m. 5	Peptide (Pe)	0.00110	-0.0136	0.1019	188.0714	2.198778	380
22	m. 5	Phen. glyco. (Pg)	0.00349	-0.1477	0.1581	623.1272	5.055600	506
32	m. 5	Phen. glyco. (Pg)	-0.00083	0.0498	-0.1267	507.1149	5.435600	638
54	m. 5	Peptide (Pe)	0.00129	0.0416	-0.0698	351.2153	6.034822	613
87	m. 5	Peptide (Pe)	0.00284	0.0082	-0.1526	1455.421	9.179689	57
96	m. 5	Phospholipid (PP)	0.00240	-0.0021	0.0564	377.2674	11.743289	691
97	m. 5	Lipid (Li)	0.00167	0.0404	-0.0329	279.2328	11.961267	834
102	m. 5	Lipid (Li)	0.00128	0.0511	-0.0726	281.2485	13.161200	884
124	m. 5	Peptide (Pe)	0.00173	0.0786	-0.1331	1099.6413	16.987733	583
49	m. 6	Alkaloid (Al)	0.00323	0.0532	0.1335	445.369	5.779267	829
98	m. 6	Saponin (Sa)	0.00015	-0.0269	-0.0464	607.2933	12.283867	483
107	m. 6	Phospholipid (PP)	-0.00005	-0.0122	-0.0705	593.2777	14.143022	468
108	m. 6	Sterol (St)	-0.00004	-0.0533	-0.0551	621.3084	14.628844	496
110	m. 6	Peptide (Pe)	0.00014	-0.0388	-0.0504	635.3816	15.485111	601
121	m. 6	Phospholipid (PP)	-0.00057	-0.0485	-0.0593	607.2933	16.585889	483
126	m. 6	Phospholipid (PP)	-0.00031	-0.0179	-0.0777	621.3084	17.422600	496
132	m. 6	Peptide (Pe)	-0.00042	-0.0410	-0.1018	635.3816	18.238778	601

66	m. 7	Saponin (Sa)	-0.00238	0.0110	-0.0479	1087.5707	6.218867	525
68	m. 7	Saponin (Sa)	0.00025	-0.0381	0.0862	943.529	6.721333	561
74	m. 7	Saponin (Sa)	0.00259	-0.0129	0.0691	585.4277	7.205911	731
75	m. 7	Saponin (Sa)	0.00227	-0.0067	0.0576	907.5367	7.203356	591
76	m. 7	Saponin (Sa)	0.00190	0.0480	0.0452	1069.5625	7.218822	526
79	m. 7	Saponin (Sa)	-0.00375	-0.0688	-0.0314	923.5027	7.572822	544
81	m. 7	Phospholipid (PP)	0.00063	0.0602	-0.0630	277.2167	8.279133	782
82	m. 7	Phospholipid (PP)	0.00058	0.0288	-0.0403	317.209	8.321022	659
31	m. 8	Phen. glyco. (Pg)	0.00117	-0.0678	-0.1842	684.2517	5.347200	368
40	m. 8	Saponin (Sa)	0.00123	0.1758	0.0983	1119.5627	5.806133	503
43	m. 8	Saponin (Sa)	0.00055	0.0050	0.0909	973.504	5.631911	518
46	m. 8	Saponin (Sa)	0.00093	0.0569	-0.2069	989.4993	5.818644	505
47	m. 8	Saponin (Sa)	0.00173	-0.1150	0.0430	843.4409	5.873844	523
52	m. 8	Saponin (Sa)	0.00121	-0.0305	0.1098	973.504	6.013467	518
56	m. 8	Saponin (Sa)	0.00285	-0.0381	0.0958	827.4455	6.058733	538
16	m. 9	Peptide (Pe)	-0.00141	-0.0005	0.0386	141.0881	3.049089	624
19	m. 9	Alkaloid (Al)	0.00186	0.0813	0.0131	422.1973	4.658444	467
72	m. 9	Peptide (Pe)	-0.00253	0.0377	-0.0505	1170.1459	7.037956	125
73	m. 9	Saponin (Sa)	-0.00558	-0.0807	0.0211	402.1992	7.065644	495
125	m. 9	Peptide (Pe)	-0.00057	0.1016	0.1027	584.4235	16.991733	725
181	m. 9	Halogen (Ha)	-0.00116	0.0197	-0.0283	127.1233	27.473089	970
117	m. 10	Peptide (Pe)	0.00509	0.0172	0.0481	595.2837	16.515511	477
118	m. 10	Peptide (Pe)	0.00425	0.0036	0.0906	613.4849	16.515178	790
137	m. 10	Sterol (St)	0.00234	-0.0163	0.0588	335.2595	18.765067	774
138	m. 10	Phospholipid (PP)	0.00259	-0.0120	0.0542	613.4849	18.837933	790
150	m. 10	Phospholipid (PP)	0.00178	-0.0083	0.0982	775.535	20.840600	690
39	m. 11	Saponin (Sa)	0.00214	-0.1306	0.0287	527.2482	5.672422	471
176	m. 11	Phospholipid (PP)	0.00557	-0.0410	0.0270	861.4798	26.956867	557
178	m. 11	Phospholipid (PP)	0.00338	-0.0390	-0.0627	835.5451	27.192933	652
179	m. 11	Phospholipid (PP)	0.00517	-0.0743	-0.0850	834.5424	27.162333	650
20	m. 12	Saponin (Sa)	0.00021	0.0792	0.1608	409.1823	4.755400	313
21	m. 12	Phen. glyco. (Pg)	-0.00217	-0.0762	-0.1660	481.2056	4.897222	427
24	m. 12	Peptide (Pe)	0.00115	0.0097	0.1753	374.1461	5.014222	390
25	m. 12	Saponin (Sa)	0.00127	0.0686	-0.1764	475.2191	5.100889	461
48	m. 12	Peptide (Pe)	0.00290	0.0146	-0.1820	728.3151	5.887111	433
60	m. 12	Saponin (Sa)	0.00040	-0.1856	0.3557	959.5744	6.105156	599
116	m. 12	Peptide (Pe)	0.00237	-0.0299	0.0653	959.5744	16.460289	599
147	m. 12	Peptide (Pe)	0.00043	0.0889	0.0042	965.6208	20.478333	643
26	m. 13	Phen. glyco. (Pg)	-0.00424	0.1468	0.0130	683.1501	5.126178	215
51	m. 13	Saponin (Sa)	0.00026	-0.0548	0.0378	915.4514	5.947444	659
61	m. 13	Saponin (Sa)	0.00113	0.0149	-0.0740	351.2153	6.255356	613
88	m. 13	Lipid (Li)	0.00097	0.0176	-0.0429	383.2564	9.921756	669
105	m. 13	Lipid (Li)	-0.00173	-0.0380	-0.0770	613.4839	13.432956	790
158	m. 13	Phospholipid (PP)	0.00100	0.0318	-0.0148	816.6582	21.926822	806
145	m. 14	Peptide (Pe)	0.00156	0.0153	0.2906	939.5958	19.884289	634
152	m. 14	Phospholipid (PP)	-0.00445	0.1972	-0.1885	941.6201	21.136822	659
156	m. 14	Sterol (St)	-0.00212	-0.0755	0.1265	427.3947	21.699578	924

TABLE S2 Compounds and coefficients from lasso regression allowing for possible effects of all individual compounds (as in Figure 3) as well as all pairwise interactions among compounds. Listed here are interactions selected by lasso regression, using binomial and Gaussian regressions for survival and adult weight, respectively (with units on those scales, as in Table S1). Results for development time are not shown here as none of the potential interactions for development time were selected by lasso regression.

Survival regression		Weight regression			
Compounds	Coefficient	Compounds	Coefficient	Compounds	Coefficient
cpd.9	-0.080	cpd.25	-0.085	cpd.43 x cpd.47	-0.030
cpd.122	0.067	cpd.49	0.0081	cpd.49 x cpd.138	0.051
cpd.154	0.0078	cpd.5 x cpd.7	-0.10	cpd.55 x cpd.79	-0.025
cpd.5 x cpd.6	-0.042	cpd.5 x cpd.9	-0.059	cpd.60 x cpd.65	0.016
cpd.5 x cpd.7	-0.0095	cpd.8 x cpd.11	-0.018	cpd.60 x cpd.79	-0.048
cpd.6 x cpd.34	-0.024	cpd.12 x cpd.152	0.019	cpd.60 x cpd.132	-0.080
cpd.6 x cpd.79	-0.11	cpd.14 x cpd.91	0.11	cpd.65 x cpd.133	-0.22
cpd.7 x cpd.133	0.078	cpd.18 x cpd.73	-0.18	cpd.85 x cpd.161	0.16
cpd.19 x cpd.54	-0.0063	cpd.18 x cpd.79	0.0048	cpd.86 x cpd.141	0.052
cpd.21 x cpd.60	-0.038	cpd.19 x cpd.85	-0.024	cpd.88 x cpd.118	0.063
cpd.45 x cpd.54	0.00024	cpd.19 x cpd.124	0.013	cpd.91 x cpd.122	-0.062
cpd.48 x cpd.142	0.019	cpd.23 x cpd.24	-0.011	cpd.95 x cpd.175	-0.011
cpd.61 x cpd.82	-0.034	cpd.25 x cpd.60	0.086	cpd.105 x cpd.125	0.024
cpd.62 x cpd.79	-0.010	cpd.25 x cpd.117	-0.019	cpd.105 x cpd.152	-0.090
cpd.83 x cpd.164	-0.017	cpd.25 x cpd.145	-0.078	cpd.117 x cpd.149	-0.10
cpd.99 x cpd.109	-0.039	cpd.32 x cpd.66	-0.37	cpd.117 x cpd.156	0.047
cpd.152 x cpd.174	-0.18	cpd.34 x cpd.94	0.00025	cpd.121 x cpd.140	0.25
		cpd.35 x cpd.49	-0.062	cpd.125 x cpd.148	0.0035
		cpd.41 x cpd.179	0.144	cpd.124 x cpd.176	0.13

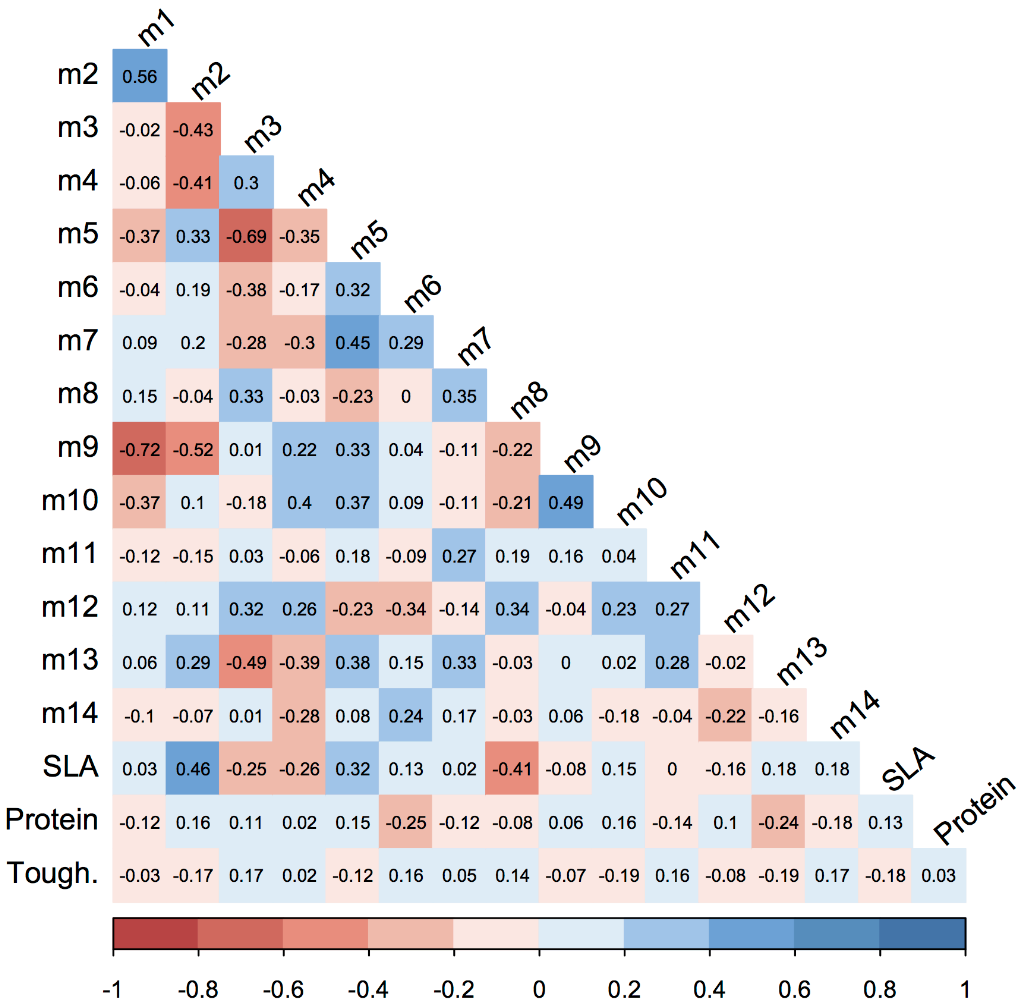

FIGURE A1 Pairwise correlations between phytochemical modules (represented by eigenvectors m1, m2, etc.) and plant traits specific leaf area (SLA), protein and leaf toughness. Values shown are Pearson product-moment correlation coefficients.

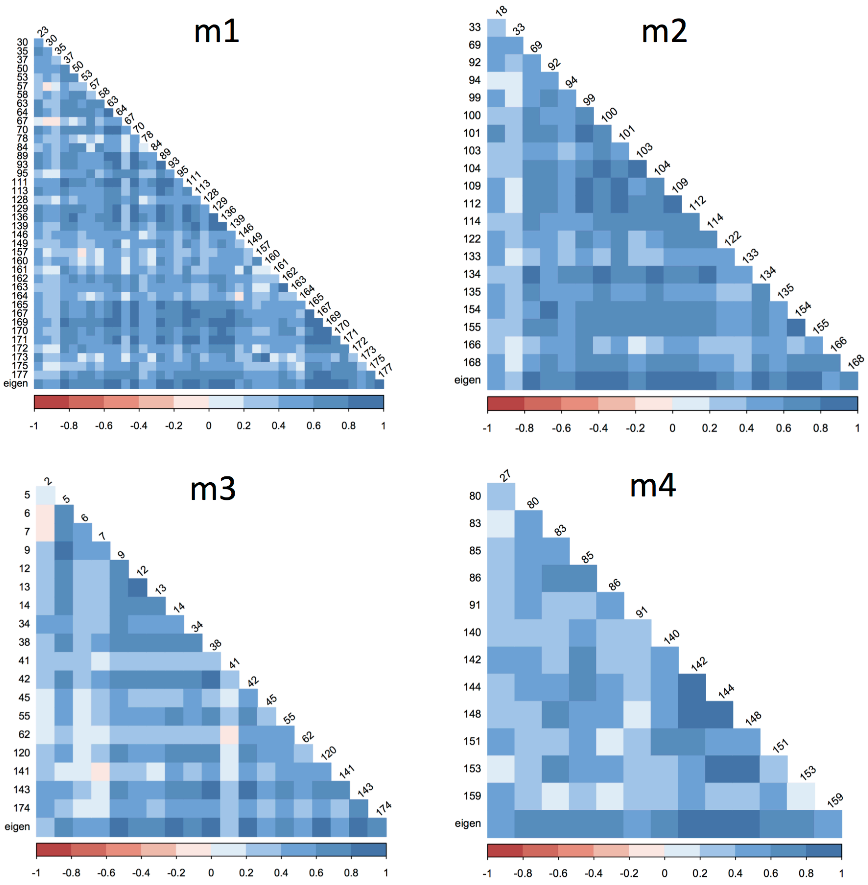

FIGURE A2a Pairwise correlations (as in Figure A1), but among compounds within modules m1 to m4. The bottom row in each graph shows correlations among individual compounds and the eigenvector used in other analyses for the given module.

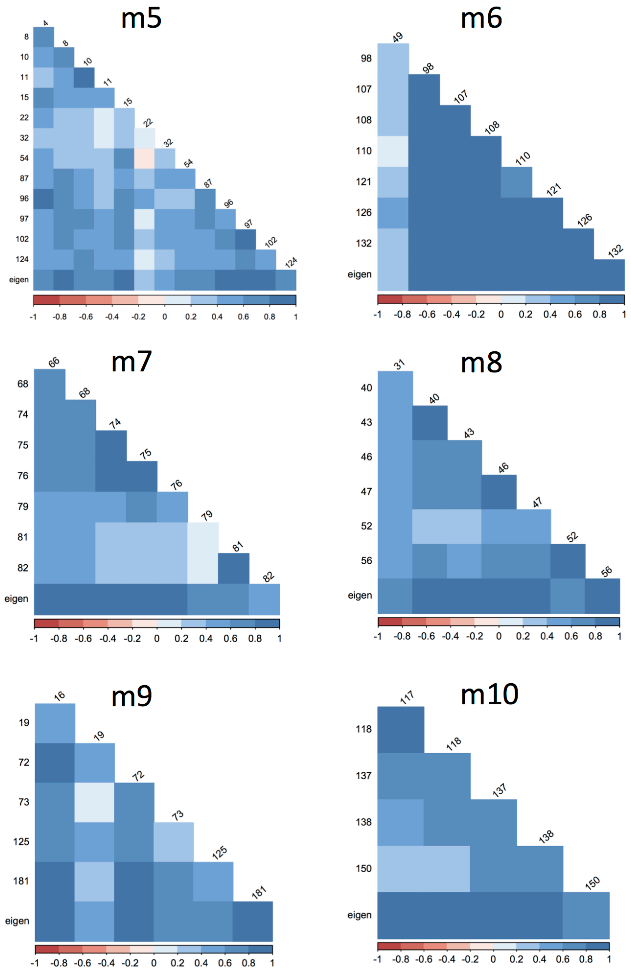

FIGURE A2b Pairwise correlations (as in Figure A1), but among compounds within modules m5 to m10. The bottom row in each graph shows correlations among individual compounds and the eigenvector used in other analyses for the given module.

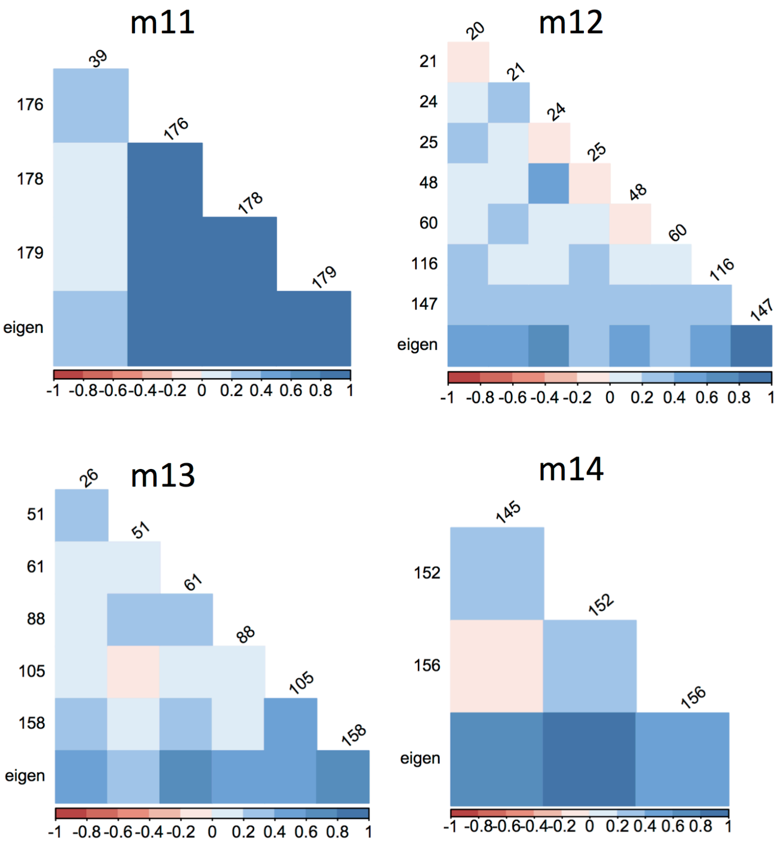

FIGURE A2c Pairwise correlations (as in Figure A1), but among compounds within modules m11 to m14. The bottom row in each graph shows correlations among individual compounds and the eigenvector used in other analyses for the given module.

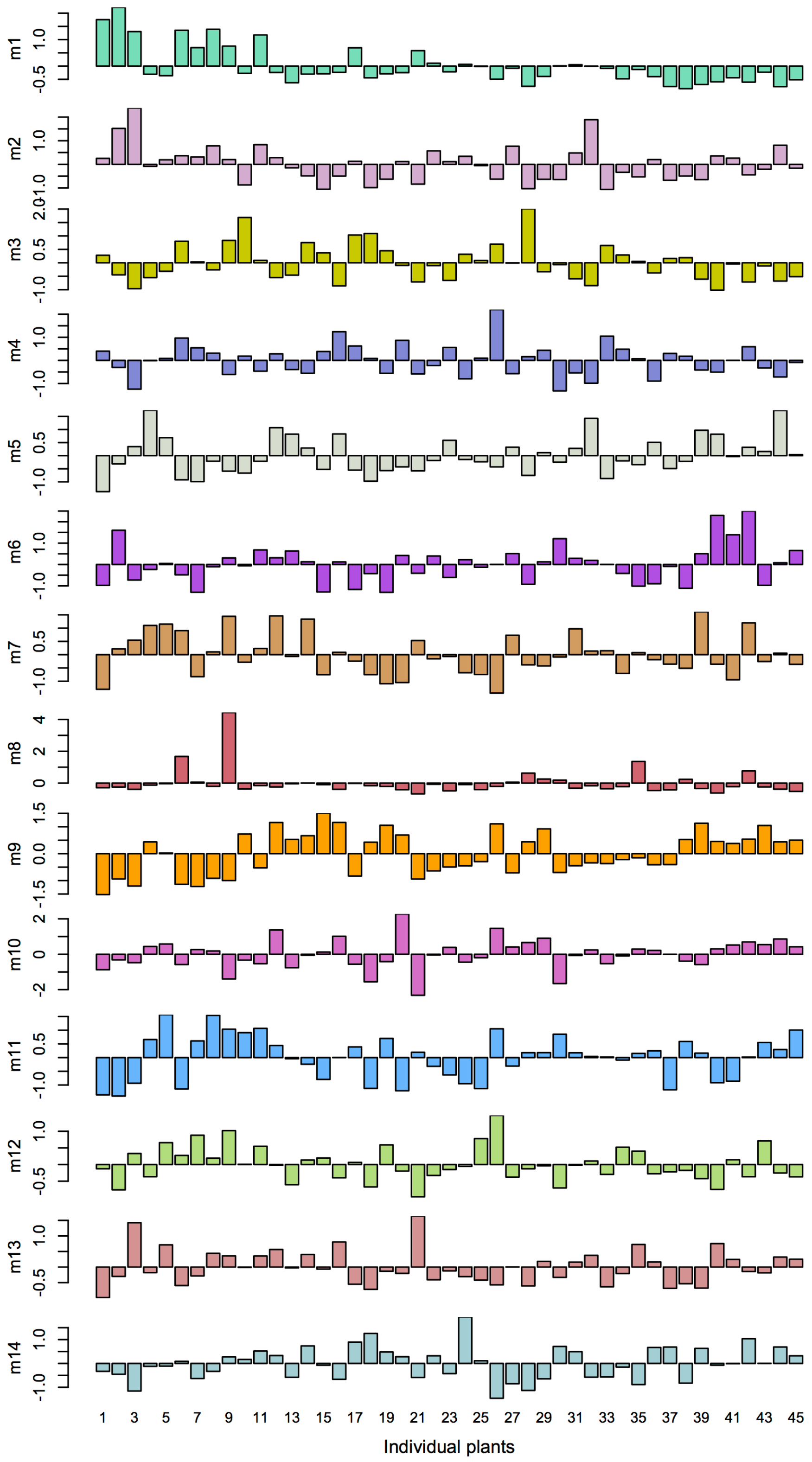

FIGURE A3 Visualization of variation among plants (bars) in phytochemical modules. Each bar is the average of z-scores for compounds comprising a given module. Colors correspond to modules as in Figure 1. The order of plants (along the x-axis) is arbitrary.

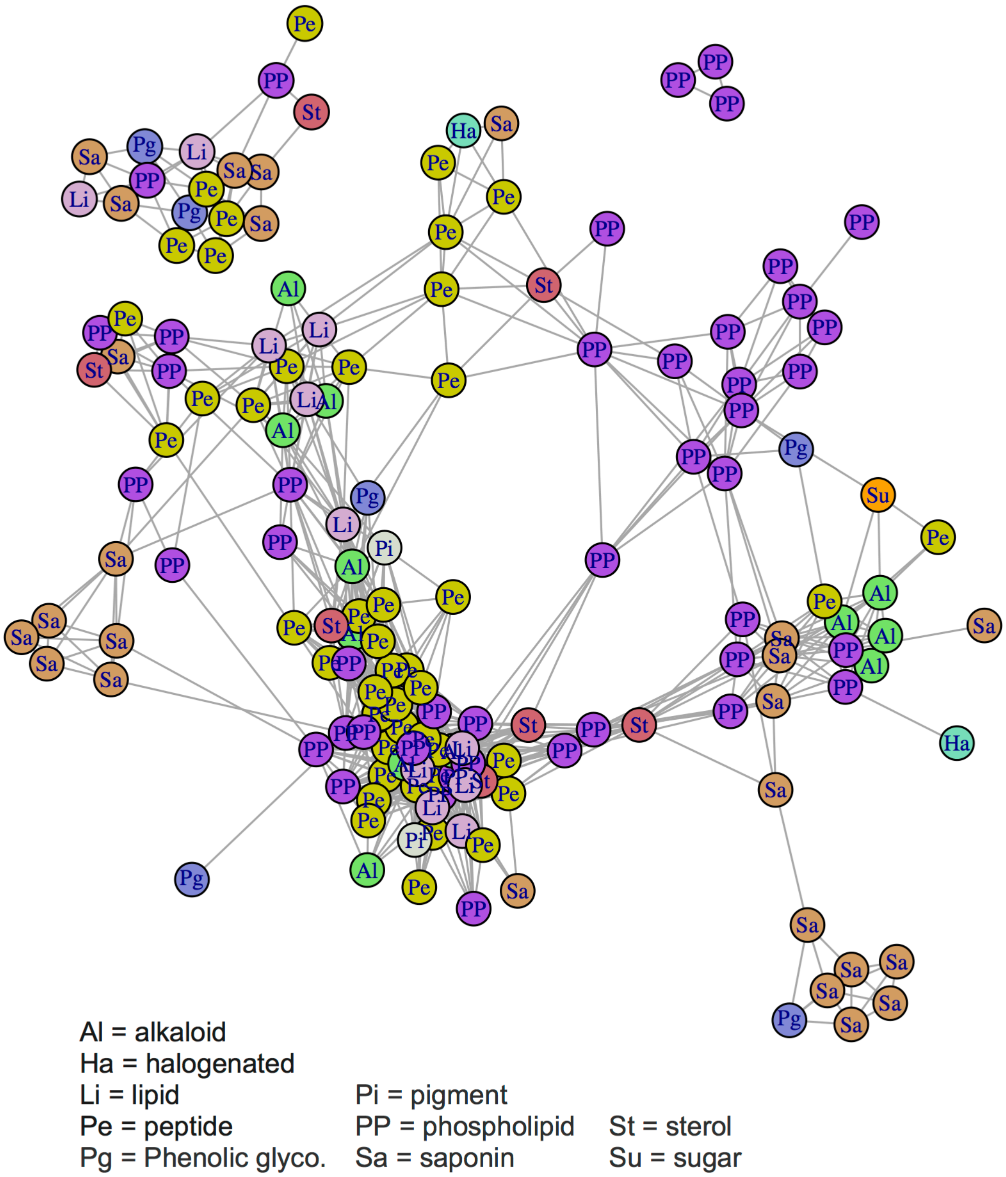

FIGURE A4 Illustration of correlational structure among compounds, as in Figure 2, but here color coded by compound class identities instead of by module assignment.

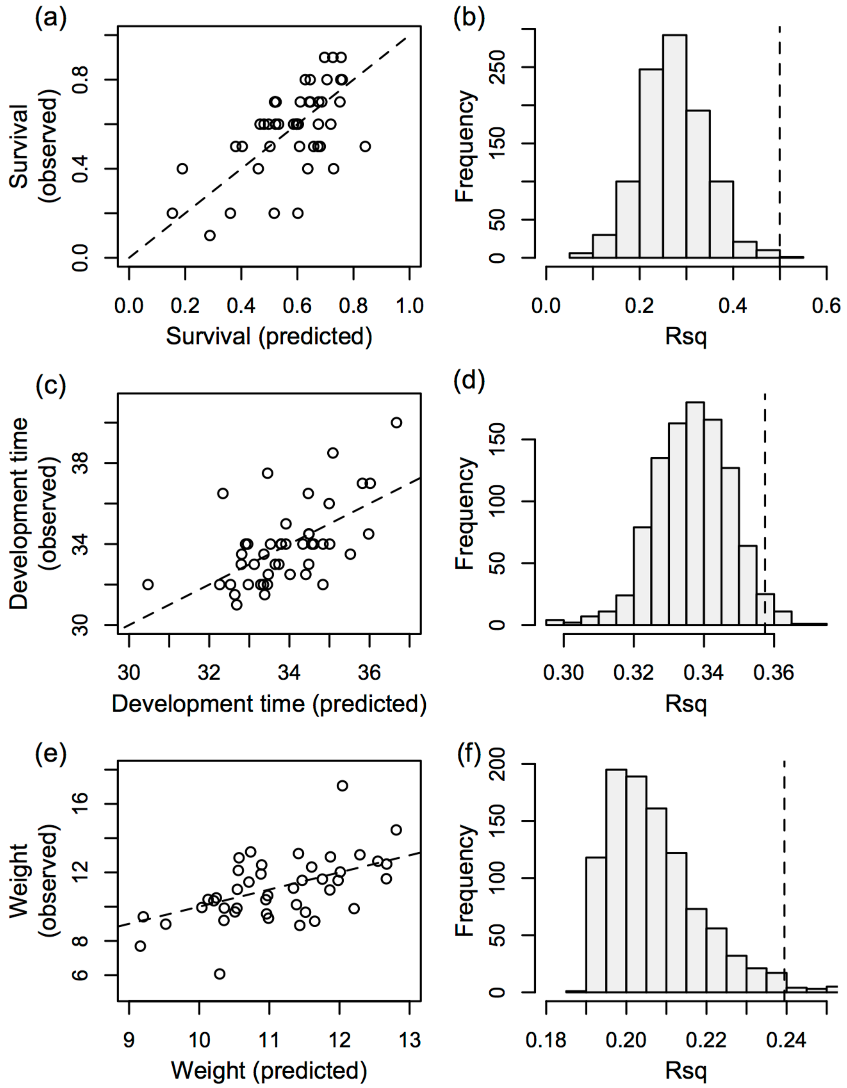

FIGURE A5 Results from cross-validation (a, c, e) and resampling analysis (b, d, f) of Bayesian regressions reported in Table 1. In the observed vs predicted plots (a, c, e), each dot represents the observed and predicted performance of caterpillars associated with individual plants, which was the level at which cross-validation was conducted (i.e., one plant was left out of each iteration). In the resampling analyses (b, d, f), "modules" were constructed based on randomly assembled collections of compounds selected to match the structure of the initial analyses. For example, the survival regressions included modules 2, 3, 9, 10, and 11 (Table 1). Because m2 includes 21 compounds, for one iteration of the resampling analysis 21 compounds were randomly selected, from which the first eigenvector was taken for use in the analysis (and same for the other modules). From each of 1000 randomly-created sets of modules, R^2 values were retained and summarized in plots (b, d, f), along with dashed lines representing the R^2 values of empirical collections of compounds and associated eigenvectors. In all cases, empirical models outperformed all but a tiny fraction of simulated models. The fraction of the simulated models with R^2 values greater than the empirical models are as follows: 0.007 (b), 0.027 (d), and 0.023 (f).

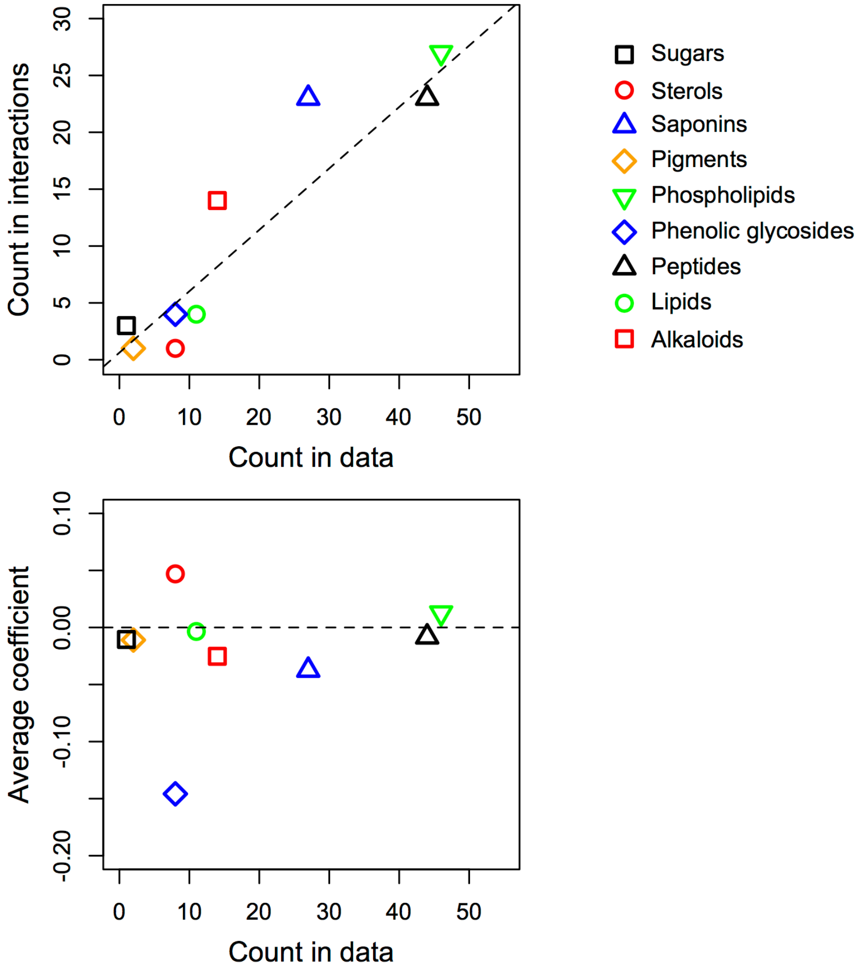

FIGURE A6 Additional details on interactions by compound class. Top panel shows the number of times that compounds of a certain class appear in pairwise interactions (as in appendix Table S2) as a function of the total numbers of those compounds in different classes ("Count in data"). Classes that are represented by a greater number of distinct compounds in the total dataset are, in general, more likely to be observed in pairwise interactions: the dotted line shows the expected frequency of observation given random sampling from the total pool of compounds. Saponins and alkaloids are overrepresented in interactions, appearing in 44 and 41% (respectively) more pairwise interactions than expected by chance. Bottom panel shows average interaction coefficients for all interactions involving compounds of particular classes: most pairwise interactions are relatively small in magnitude, with the exception of interactions involving phenolic glycosides, that tend to be strongly negative. For both panels, interactions are considered from analyses of caterpillar survival and weight; no interactions were detected for development time (Supporting Information Table S2).

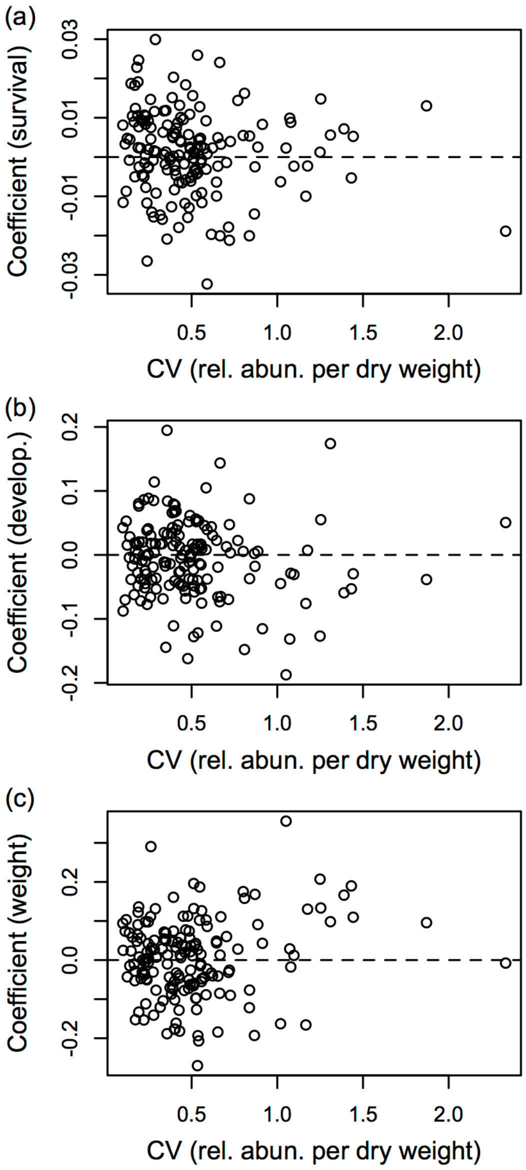

FIGURE A7 Compound specific effects vs coefficients of variation for individual compounds. The compound specific effects based on ridge regression are the same as those listed in Table S1.

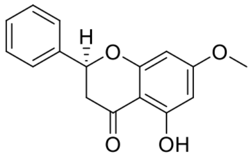

Pinostrobin
Chemical Formula: $C_{16}H_{14}O_4$
Exact Mass: 270.0892
RMD: 330

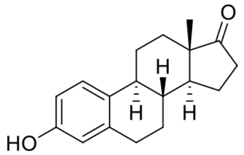

Estrone
Chemical Formula: $C_{18}H_{22}O_2$
Exact Mass: 270.1620
RMD: 600

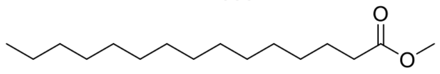

Methyl palmitate
Chemical Formula: $C_{17}H_{34}O_2$
Exact Mass: 270.2559
RMD: 948

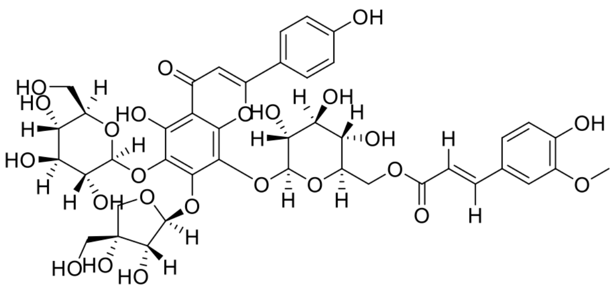

Apigenin glycoside
Chemical Formula: $C_{42}H_{46}O_{24}$
Exact Mass: 934.2379
RMD: 255

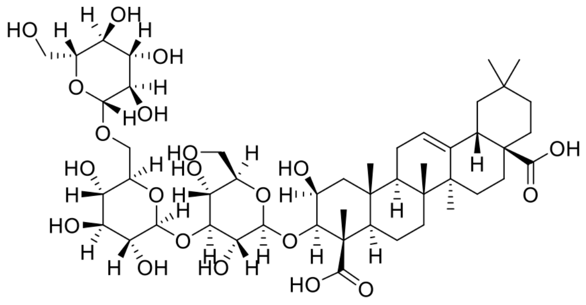

Medicagenic acid glycoside
Chemical Formula: $C_{48}H_{76}O_{21}$
Exact Mass: 988.4879
RMD: 494

Figure A8. Relative mass defect of natural products. **Left:** A series of isobaric molecules (by nominal mass) which have increasing RMD as %H increases. **Right:** Characteristic metabolites from *M. sativa* which can be classified by RMD.
